# Personality profile of amateur team handball referees

**DOI:** 10.1007/s12662-022-00837-8

**Published:** 2022-07-14

**Authors:** Marcus Dodt, Frowin Fasold, Daniel Memmert

**Affiliations:** 1 Department of Computer Science in Sports and Team/Racket Sport Sciences, German Sport University Cologne, Am Sportpark Müngersdorf 6, 50933 Cologne, Germany; 2 Department of Cognitive and Team/Racket Sport Research, German Sport University Cologne, Am Sportpark Müngersdorf 6, 50933 Cologne, Germany; 3 Heimkerweg 29, 58706 Menden, Germany

**Keywords:** Sports psychology, Big Five Inventory 2, Team sports, Refereeing, Leisure sports

## Abstract

Referees play a central role in competitive sport. Particularly in amateur sport, referees contribute significantly to making participation in sport possible for the masses. However, considering that every referee’s career starts at the grassroots level, it is very surprising that there has been no research on the personality traits of amateur referees so far. The current state of research indicates that personality is an essential component of the requirement profile of referees. Personality has been associated with job performance, particularly with regard to resilience and coping with pressure. Personality also affects effective game management in terms of influencing actions in the game in a preventative and proactive manner. This study, therefore, examines the personality profile of amateur handball referees (*n* = 582) for the first time using the German version of the Big Five Inventory 2 (BFI-2). Current data from German handball referees at the expert level and the German general population were used to compare and discuss the results. Except for lower scores in the domain of extraversion and the facets of sociability and energy level as well as a higher score in the facet of aesthetic sensitivity, amateur referees did not differ significantly from expert referees. In relation to the general population, the results indicate that handball referees, regardless of performance level, have higher scores in assertiveness, emotional stability, and responsibility. Our findings create awareness of personality traits in handball refereeing and illustrate the applied relevance of personality research, e.g., for coaching or recruitment activities.

## Introduction

### Personality profile of amateur team handball referees

Referees play a vital role in almost every team sport. Without their engagement, sport competition would not be possible. This applies to all divisions, from professional leagues to the lower leagues (Balch & Scott, 2007; Phillips & Fairley, 2014). Although referees from all divisions have one thing in common—namely to make certain that the rules of the game are followed—it should be considered that the refereeing style that is needed at each level of performance is different (MacMahon et al., 2015).

At the elite level, decision-making is much more demanding due to the higher speed and dynamics. In addition, creating flow and exercising control depends to a large extent on the referee’s thorough understanding of the game (Mascarenhas, Collins, & Mortimer, 2005; Slack, Maynard, Butt, & Olusoga, 2013). A mindful balance and interpretation of the rules is particularly crucial in open invasion games like handball and contributes to increasing the (media) attractiveness of the sport (MacMahon et al., 2015). Thus, referees are an important part of the professional “staging,” who must always be masters of the situation without unnecessarily interrupting the flow and spectacle of the game.

At the non-professional level, in turn, beginners and amateurs require a more educational way of officiating which creates a safe playing and fun environment. Consequently, the task of the referees at this level of performance is also to make the sporting activity interesting and thus encourage participation and engagement (Płoszaj, Firek, & Czechowski, 2020). These so-called bread and butter referees (MacMahon et al., 2015, p. 15) play a major role in ensuring sporting activity at the mass sports level and in conveying the rules and the spirit of the game to young people, thus fulfilling an important social task (Andersson, 2019; Firek, Płoszaj, & Czechowski, 2020). Despite all the differences between the league-dependent refereeing styles, ensuring compliance with the rulebook places task-specific demands on referees. In this context, among other requirements, the personality of referees is considered significant for performance of the job (Mascarenhas et al., 2005).

The trait theory of personality proposes that stable characteristics predispose an individual to behave consistently across different situations (see Boyle, 2010 for an overview of the approaches of Allport, Cattell, and Eysenck). Following this consideration, good refereeing depends to a large extent on the personality traits of a referee. No matter how well a referee knows the rules of the game, certain traits can help them communicate more effectively with players and coaches and let them know that they are in control of the game. This includes how they behave and relate to others prior to, during, and after competition, and how they present themselves as a referee. Personality can also have an impact on an individual’s ability to make and execute decisions (Byrne, Silasi-Mansat, & Worthy, 2015), to deal with stress (Kaiseler, Polman, & Nicholls, 2012), but also to be patient and resilient in the face of aggressive behavior (Devís-Devís, Serrano-Durá, & Molina, 2021; Wolfson & Neave, 2007). Despite these findings and the question already raised as to which psychological characteristics are required for officiating (Livingston et al., 2017), it is surprising that the personality profile of amateur referees has been almost completely neglected in research to date.

In the context of recruitment, personality or personality assessment has become established in recent years in professional (Barrick & Mount, 1991) and academic (Poropat, 2009) fields of application and has also found its way into sport (Steca, Baretta, Greco, D’Addario, & Monzani, 2018). Recent research provides support for the use of personality profiles in the prediction of athletic success, thus contributing significantly to the process of talent identification (Gee, Marshall, & King, 2010). This talent stage is also particularly crucial in referee recruitment. MacMahon et al. (2015) adapted the FTEM (Foundations, Talent, Elite, Mastery) framework (Gulbin, Croser, Morley, & Weissensteiner, 2013) and developed a framework to describe the development pathways of officials. Within this framework, the talent stage is considered “the gateway to elite performance, to maintenance of regular participation, or to drop out” (MacMahon et al., 2015, p. 14). For the amateur sector, therefore, this crossroad represents a trouble area in the recruitment of referees. As an individual’s personality can be considered a decisive factor in whether he or she starts officiating or succeeds in the profession (e.g., Mascarenhas et al., 2005; Ridinger, Warner, Tingle, & Kim, 2017; Weinberg & Richardson, 1990), it seems reasonable to investigate the personality traits of handball referees at amateur level in more detail. Especially in the transition phase from casual and often informal involvement to regular involvement, comparative values from the amateur sector are lacking but could provide additional guidance in the decision-making process of potential referee candidates (“Do I have the ‘right personality’ for this job?”).

So far, no research results are available on the personality of amateur referees in handball. Therefore, the central questions of the present study are (a) which personality traits do referees at amateur level exhibit, (b) how do amateur referees differ from referees at the expert level, and (c) how can the results be classified in relation to the general population. Thus, our study aims at closing the existing knowledge gap, creating awareness of the personality traits of handball referees in the amateur sector, and providing the fundament for further considerations and concepts, e.g., in optimizing the recruitment process or coaching activities.

The most widely accepted and frequently used instrument for measuring personality trait structure is the five-factor model (Costa & McCrae, 1992). The model explains the most important characteristics of human behavior and consists of five independent personality traits that remain relatively stable throughout life: openness, conscientiousness, extraversion, agreeableness, and neuroticism. Research studies have indicated that personality is associated with regular exercise (Rhodes & Pfaeffli, 2012; Rhodes & Smith, 2006) and athletic success (Piedmont, Hill, & Blanco, 1999). Thus, there is good reason to expect that personality could also be a predictor of engagement in officiating.

The remainder of this study is structured as follows: first, we give an insight into the current state of research and present the theoretical framework including a description of the five-factor model. Second, in the “Methods” section, we describe the participants, the materials, the procedure, and the data evaluation in more detail. Third, we present the results in two subsections, one comparing amateur referees to expert referees and the other comparing amateur referees to the general German population. Finally, the article concludes with a discussion of the results, including limitations and future perspectives.

### State of research

Despite their importance for the functioning of sporting competitions, referees are an under-researched subpopulation in the sports psychology literature. One of the first topics investigated in this area was the sources of stress affecting refereeing and how this stress is dealt with (e.g., Rainey, 1995; Taylor & Daniel, 1987). The most stressful situations for referees occur when the referee instructor was present at the game, when there were mistakes in the officiating mechanics, when a foul was incorrectly called, when there was an injury, when there were protests by the players or coaches, or when the instant replay was used (Anshel, Sutarso, Ekmekci, & Saraswati, 2014; Anshel & Weinberg, 1995; Kaissidis-Rodafinos & Anshel, 2000; Kaissidis-Rodafinos, Anshel, & Sideridis, 1998). However, only about 5% of situations that arise in a game cause a high level of stress, while the normal course of the game leads to a low or medium level (Rainey & Winterich, 1995; Stewart, Ellery, Ellery, & Maher, 2004).

More recently, studies have focused on aspects that influence refereeing decisions, such as public pressure (Di Corrado, Pellarin, & Agostini, 2011; Myers & Balmer, 2012; Unkelbach & Memmert, 2010), the decision-making process (Burnett, Bishop, Ashford, Williams, & Kinrade, 2017; Neville, Salmon, & Read, 2017; Souchon et al., 2016), or referee self-efficacy (Lirgg, Feltz, & Merrie, 2016; Myers, Feltz, Guillén, & Dithurbide, 2012). During the COVID-19 pandemic, for example, the absence of spectators could be used as a natural experiment and showed that in the absence of spectators, the increased sanctioning of guest teams disappeared (Wunderlich, Weigelt, Rein, & Memmert, 2021). Regarding handball referees (who always work with the same partner), Diotaiuti, Falese, Mancone, and Purromuto (2017) could confirm the role of perceived couple efficacy as a predictor of the perception of self-efficacy. Officiating as a team or couple for a longer period of time also leads to a better relationship among the couple, more stability in the decision-making process, and fewer disagreements.

However, there is little research on the personality profile of referees. A review of the few studies published on this issue suggests that the results are inconclusive, probably due to the different methods and instruments used (Gomà-i-Freixanet, Pla-Cortés, & Avilés-Antón, 2020). Thus, studies in the 1970s and 1980s (e.g., Alker, Straub, & Leary, 1973; Dale, 1976; Ittenbach & Eller, 1988; Quain & Purdy, 1988; Sinclair, 1975) used the California Psychological Inventory (Gough, 1957) for personality assessment. These studies suggest that dominance or related facets, such as self-acceptance or leadership, would define the characteristics of referees with higher levels of professional experience, and that more experienced referees would tend to score higher in extraversion compared to those with less experience.

More recently, studies have been published using the questionnaires developed by Costa and McCrae (NEO PI‑R and NEO-FFI; Costa & McCrae, 1992). For example, Balch and Scott (2007) found that referees in volleyball, hockey, and wrestling scored significantly higher in extraversion than the control group of non-officials. Winters (2010) concluded that major college baseball umpires compared to umpires from lower competition levels showed lower levels of gregariousness and fantasy and a higher level of modesty. Further, Pla-Cortés, Gomà-i-Freixanet, and Avilés-Antón (2015) compared Spanish expert basketball referees with the Spanish general population and found that referees scored significantly higher in neuroticism and lower in open-mindedness, agreeableness, and conscientiousness. Gomà-i-Freixanet et al. (2020) ascertained in their long-term study that Spanish basketball referees who have reached the elite level displayed greater competence and assertiveness, and were more self-assured, efficient, and organized. Furthermore, they were more dominant and possessed better leadership skills than their colleagues who have not promoted in their career. Another study by McCarrick, Wolfson, and Neave (2019) suggested that soccer referees at the highest level possess critical personality characteristics with respect to mental toughness, locus of control, assertiveness, and social comparison than intermediates and amateurs. Ultimately, only one study is known to date that deals with the personality traits of handball referees: Dodt, Fasold, and Memmert (2022) found that handball referees at the expert level scored higher values in extraversion, agreeableness, and conscientiousness and a lower value in negative emotionality compared to the German general population.

This review of the literature on referee personality profiles shows that the personality values collected in these studies are always related to a different control group: either referees of different performance levels are compared to each other (e.g., Gomà-i-Freixanet et al., 2020) or referees are compared to standard values of the general population (e.g., Pla-Cortés et al., 2015). In this study, both approaches are incorporated and combined by putting the findings in relation to handball referees at the expert level as well as the German general population. Following this methodological approach, the results of the present study can be better classified, and it can be revealed whether there might be differences between amateurs and experts *and* between amateurs and the general population. This study can thus be seen as an extension of the work of Dodt et al. (2022), with the advantage of a broader (and more meaningful) database on the personality profile of handball referees.

### Theoretical framework and the present study

The present study aims at investigating the personality profile of handball referees in more depth and amplifying the current state of research with a clear focus on amateur referees. So far, only one study on the personality traits of referees at the elite level is known (Dodt et al., 2022). Since the research presented suggests that differences exist in certain personality domains or facets both between referees of different performance levels (Gomà-i-Freixanet et al., 2020; McCarrick et al., 2019; Winters, 2010) and between referees and a norm sample (Dodt et al., 2022; Pla-Cortés et al., 2015), it is assumed that this could also apply to handball referees. Although the referees from the aforementioned studies came from different sports (basketball, soccer, and handball), they are comparable in terms of their task demands (i.e., many actions to evaluate and a high level of communication). In order to reveal potential differences also among handball referees, it therefore seems reasonable to compare the results of the present study with the existing data on expert referees as well as with norm values of the German general population.

Given the scarcity of literature, especially for amateur referees, and the lack of scientific consensus (e.g., contrary findings in neuroticism: Dodt et al., 2022; Pla-Cortés et al., 2015), this study is unique in three respects: (a) this study is the first to put amateur handball referees at the heart of research interest; (b) the large sample size of this study *(n* = 582) allows description of the personality profile of amateur referees as precisely as possible; (c) by comparing the data with those of expert referees and the German general population, a comprehensive picture of the personality profile of handball referees can be created. These points are essential to approaching the current challenges in handball refereeing from a sport psychological perspective. The findings of this study are intended to provide initial insights to better understand the personality of amateur referees, from which further research or initial operational measures can be derived.

The five-factor model used by Dodt et al. (2022) also provides the theoretical foundation for this study with amateur referees. In the following section, the model is first introduced and the assumptions underlying this study are then presented.

**Fig. 1:**
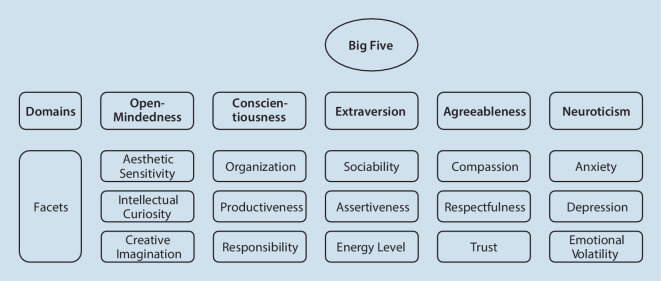
Big Five Inventory 2 domains and facets

The five-factor model (Costa & McCrae, 1992) is a hierarchical model that captures the Big Five personality (see Fig. 1) traits of open-mindedness, conscientiousness, extraversion, agreeableness, and negative emotionality with three facets each (Soto & John, 2017). It is considered a “universal” personality model (John, Naumann, & Soto, 2008; McCrae & Costa, 2008) and has been tested in several studies with regard to its accuracy in different countries and cultures (cf. Ostendorf & Angleitner, 1992). The model has also been applied in the sporting setting, but never before with amateur handball referees. Since its suitability has been proven in the context of sports and especially in the specific application with referees (Dodt et al., 2022; Gomà-i-Freixanet et al., 2020, Pla-Cortés et al., 2015), we consider this model to be highly adequate for the present study.

The openness dimension distinguishes between those who are open to new experiences (curious, creative, and imaginative) and those who prefer routine (conventional, uncreative, and unimaginative); the conscientiousness dimension distinguishes those who are conscientious (hardworking, organized, and reliable) from those who are lackadaisical (unreliable, lazy, and careless); the extraversion dimension distinguishes between those who are extraverted (sociable, outgoing, and active) and those who are introverted (unsociable, quiet, and passive); the agreeableness dimension distinguishes between those who are compassionate (helpful, trusting, and empathetic) and those who tend to antagonize (cynical, rude, and uncooperative); and the neuroticism dimension distinguishes those who possess emotional stability (calm, even-tempered, and secure) from those who are emotionally unstable (anxious, hostile, and irritable).

Because of the limited research on referee personality, only assumptions can be made for this study. Studies comparing the personality scores of athletes at different performance levels showed lower scores for extraversion, agreeableness, and conscientiousness and a higher score for negative emotionality (lower emotional stability) in amateur athletes compared to elite athletes (e.g., Allen, Greenlees, & Jones, 2011; Egloff & Gruhn, 1996; Piedmont et al., 1999; Steca et al., 2018). However, these performance differences do not seem to be readily transferable to handball referees. A recent study with basketball referees, who are very similar to handball referees in terms of task specificity—both belong to the group of interactors (Plessner & MacMahon, 2013)—could not find any significant differences between elite and recreational levels (Gomà-i-Freixanet et al., 2020). Thus, there is no reason for the present study to assume that amateur handball referees should differ from referees at the expert level in any of the Big Five domains. However, at the facet level Gomà-i-Freixanet et al. (2020) and McCarrick et al. (2019) found that basketball or soccer referees who reached the elite level scored significantly higher assertiveness values than their colleagues from lower leagues. This could also indicate a difference in handball referees from different performance levels and thus a lower assertiveness score for amateur referees.

Nevertheless, based on this assumption that apart from possible small nuances in the facets, no performance-related differences are to be expected, significant differences are assumed in comparison to the general population. Studies with handball and basketball referees, however, do not provide consistent findings in terms of a population-based comparison. While Dodt et al. (2022) suggest that there are no differences between handball referees at the expert level and the German general population in the domains of openness and conscientiousness, Pla-Cortés et al. (2015) found lower scores for basketball referees compared to the Spanish general population. Conversely, basketball referees do not show any noticeable difference in the domain of extraversion, whereas handball referees score significantly higher than the general population. In the domains of agreeableness and neuroticism, there are opposite findings for basketball and handball referees, which show either higher or lower values compared to the norm sample.

In summary, with regard to population-based differences between amateur handball referees and the German general population, referees are expected to score similar or lower values in the domains of openness and conscientiousness and a higher value in the domain of extraversion. The assumption for the domain of extraversion can also be supported by studies that have shown that athletes generally have higher levels of extraversion than non-athletes (e.g., Egloff & Gruhn, 1996). For the domain of agreeableness, it is assumed that amateur referees do not differ from expert referees and, thus, according to the results of Dodt et al. (2022), there are also significant deviations from the general population. Finally, for the domain of negative emotionality, studies have demonstrated that athletes have greater levels of emotional stability than non-athletes (Eysenck, Nias, & Cox, 1982; McKelvie, Lemieux, & Stout, 2003; Steca et al., 2018). Furthermore, handball referees at the expert level demonstrate significantly higher emotional stability compared to the general population (Dodt et al., 2022). For the present study, the underlying assumption is that emotional stability is just as important for amateur handball referees as it is for expert referees and is, therefore, also more pronounced compared to the general population—which means a lower value for negative emotionality.

In conclusion, we note that the body of literature and the theoretical fundament is limited, in particular with regard to handball referees. The approach of the present study, therefore, aims at gaining a better understanding of the personality profile of handball referees. Nevertheless, the current state of research provides good evidence for the assumptions mentioned above. Based on these considerations, we believe that amateur handball referees do not differ significantly in their personality traits from referees at the expert level, but display significant differences compared to the general population in the domains of extraversion and negative emotionality.

## Methods

In order to examine the assumptions made above, a cross-sectional study was conducted as a self-report with German amateur handball referees. This includes referees who are active in the fourth league and below, and can therefore be assigned to the leisure sport level. For comparison and classification of the collected data, the study results of Dodt et al. (2022), who examined handball referees from the competitive sport level (*n* = 163)^1^, and Danner et al. (2019), who provide norm values for the German general population (*n* = 613), were used.

To carry out this study, we obtained the approval of the local Ethics Committee (ethics application no. 082/2020) and the German Handball Federation. The study was also conducted following the guidelines of the Declaration of Helsinki (World Medical Association, 2013).

### Participants

For this study, German amateur handball referees (*n* = 582, mean age [M_age_] = 37.9 years, standard deviation [SD] = 15.1 years, age range: 14–72 years) were interviewed during the 2019/20 season. They had a wide range of officiating experience (M = 15.6 years, SD = 12.6 years, range: 0–50 years) and a high educational level (64.4% stated that they have a university entrance qualification). 54.6% of the sample reported being the eldest child in their family, 27.8% the second eldest. Regarding fitness level, 4.5% of the referees considered themselves as athletic, 15.6% as very well trained, 32.3% as well trained, 31.4% as moderately trained, 14.1% as less trained, and 1.5% as untrained.

### Materials

The online survey consisted of the German-language scale of the Big Five Inventory 2 (BFI-2). The German translation of the BFI‑2 (Danner et al., 2019) is an adaptation of the original English version by Soto and John (2017), which in turn is based on the Big Five Model of personality (Costa & McCrae, 1992). The instrument consists of 60 items and captures the Big Five personality traits of open-mindedness, conscientiousness, extraversion, agreeableness, and negative emotionality with three facets each. The participants had the opportunity to indicate the extent to which they agree or disagree with the statements on a five-point Likert scale from 1 (strongly disagree) to 5 (strongly agree). All items were programmed as mandatory items, which means that only fully completed data records were transferred to the analysis. Therefore, a complete dataset without missing values could be evaluated.

The BFI‑2 possesses good reliability estimates, with Cronbach’s alpha between 0.77 and 0.89 (mean 0.83) for the domain values and Cronbach’s alpha between 0.57 and 0.87 (mean 0.71) for the facet values. Similar reliability values were reported by Danner et al. (2019) and Dodt et al. (2022). The domain and facet values were predominately nonnormally distributed, as assessed by the Shapiro–Wilk test, *p* < 0.05.

### Procedure

An online survey was created for this study. The mailing lists of those responsible for the respective regional associations could be used to distribute the link. After 2 weeks, a reminder was sent with the request to participate in the study. In addition, the link to the survey was posted in referee groups on social media. Particularly on Facebook, the referee community is well organized and networked in separate groups, so many referees could be reached via this channel. The survey included informed consent, which ensured that all participants agreed to the anonymous processing of data. Only fully completed questionnaires were evaluated.

### Analysis

IBM SPSS Statistics for Windows, version 25 (IBM Corp., Armonk, N.Y., USA) was used for data analysis. The data of the amateur referees were first evaluated descriptively. Because of the sufficiently large sample size and homogeneity of variances (Levene’s test yielded no significant results, all *p*-values > 0.05), the data could be analyzed despite violation of the normality assumption (Stone, 2010; Wilcox, 2013). To explore the differences in personality traits between amateur referees examined in this study and expert referees (Dodt et al., 2022) as well as the German general population (Danner et al., 2019), *t*-tests and confidence intervals (CI) for the difference between means^2^ were calculated. Cohen’s *d*_s_ values were calculated as indicators of the effect size (Cohen, 1988; Lakens, 2013). This comparison is admissible because the data were collected with the same instrument.

## Results

**Fig. 2:**
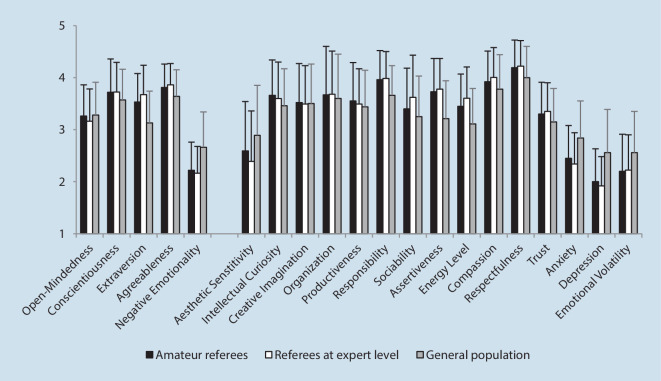
Mean values of the Big Five Inventory 2 domains and facets expressed by amateur referees compared to referees at the expert level and the German general population (error bars show standard deviations)

This section is divided into two parts and first presents the results of the comparison between amateur and expert referees (*n* = 163; Dodt et al., 2022) and second the comparison between amateur referees and the German general population (*n* = 613; Danner et al., 2019). Detailed information on descriptive and inferential statistics is reported in Tables 1 and 2 in the appendix. Figure 2 shows the mean values of all BFI‑2 domains and facets for amateur referees, expert referees, and the German general population.

### Comparison 1: amateur referees vs. referees at expert level

The assumed average value for amateur referees (M = 3.26, SD = 0.60, 95% CI [3.21, 3.31]) in the domain of open-mindedness can be confirmed. Although the value of amateur referees is descriptively higher than the value of referees at expert level (M = 3.16, SD = 0.62, CI [3.07, 3.26]), the difference cannot be statistically confirmed, *t* (743) = 1.77, *p* = 0.077, *d* = 0.15. This also applies to two associated facets (intellectual curiosity and creative imagination). One exception is the facet of aesthetic sensitivity, where amateur referees (M = 2.59, SD = 0.95, 95% CI [2.51, 2.67]) score higher than expert referees (M = 2.39, SD = 0.97, 95% CI [2.24, 2.55]), which is also statistically recognizable *t* (743) = 2.31, *p* = 0.021, *d* = 0.21.

For the domain of conscientiousness, the assumption that amateur referees (M = 3.72, SD = 0.64, CI [3.67, 3.78]) score an average to high value and do not differ from referees at expert level (M = 3.72, SD = 0.57, CI [3.63, 3.81]) could be descriptively confirmed. Statistically, the consistency of the data is also clearly visible, *t* (743) = 0.02, *p* = 0.984, *d* = 0.00. This also applies to the similarity of the facet values (all *p-*values > 0.447 and all *d*’s < 0.07).

A descriptively visible difference was found between amateur referees (M = 3.53, SD = 0.55, CI [3.48, 3.57]) and referees at the expert level (M = 3.67, SD = 0.56, CI [3.59, 3.76]) in the domain of extraversion. Therefore, the assumption that referees from lower leagues are as extroverted as their colleagues at expert level cannot be confirmed, which is also supported by the data, *t* (743) = −2.97, *p* = 0.003, *d* = −0.26. This finding also applies to the values of the facets sociability and energy level, where amateur referees obtained lower values than referees at the expert level. In contrast, amateur referees (M = 3.73, SD = 0.64, CI [3.68, 3.78]) scored almost as highly in assertiveness as their colleagues at the expert level (M = 3.78, SD = 0.59, CI [3.69, 3.88]), which is why no difference between performance levels was detected for this facet, *t* (743) = −1.06, *p* = 0.289, *d* = −0.09.

For the domain of agreeableness, the assumption that high values would be characteristic for both amateur referees (M = 3.81, SD = 0.45, CI [3.77, 3.84]) and referees at the expert level (M = 3.86, SD = 0.41, CI [3.80, 3.93]) could be confirmed. Statistically, no difference could be detected between the two groups, *t* (743) = −1.45, *p* = 0.147, *d* = −0.13. The same applies to the associated facets of compassion, respectfulness, and trust (all *p*-values > 0.119 and all *d*’s < −0.15).

Finally, the assumption that low values in negative emotionality would also be characteristic of amateur referees (M = 2.22, SD = 0.54, CI [2.17, 2.26]) was descriptively confirmed. Referees at expert level (M = 2.16, SD = 0.51, CI [2.08, 2.24]) show only minor deviations, thus a difference cannot be statistically verified, *t* (743) = 1.14, *p* = 0.254, *d* = 0.10. A similar picture was found for the facets of anxiety, depression, and emotional volatility (all *p*-values > 0.063 and all *d*’s < 0.16).

### Comparison 2: amateur referees vs. German general population

The assumption that amateur referees (M = 3.26, SD = 0.60, 95% CI [3.21, 3.31]) do not differ from the general population (M = 3.28, SD = 0.63) in the domain of open-mindedness can be confirmed. Descriptively, the values are almost identical, which is also expressed statistically, *t* (581) = −0.87, *p* = 0.385, *d* = −0.04.

For the domain of conscientiousness, the assumption that amateur referees (M = 3.72, SD = 0.64, CI [3.67, 3.78]) do not differ from the general population (M = 3.57, SD = 0.59) could not be descriptively confirmed, which is also supported by the data, *t* (581) = 5.71, *p* < 0.001, *d* = 0.24. This is also particularly applicable to the facet of responsibility. Amateur referees (M = 3.96, SD = 0.56, CI [3.91, 4.00]) score higher than the general population (M = 3.66, SD = 0.57), which is also statistically visible, *t* (581) = 12.61, *p* < 0.001, *d* = 0.52.

A descriptively visible difference between amateur referees (M = 3.53, SD = 0.55, CI [3.48, 3.57]) and the general population (M = 3.13, SD = 0.61) was also found in the domain of extraversion. Therefore, the assumption that amateur referees have a more extrovert nature can be confirmed, which is also statistically supported, *t* (581) = 17.35, *p* < 0.001, *d* = 0.72. Significant differences can also be reported for all three facets, with energy level, *t* (581) = 13.33, *p* < 0.001, *d* = 0.55, and assertiveness, *t* (581) = 19.60, *p* < 0.001, *d* = 0.81, particularly standing out with medium and large effect sizes.

For the domain of agreeableness, the assumption that amateur referees (M = 3.81, SD = 0.45, CI [3.77, 3.84]) would score higher values than the general population (M = 3.64, SD = 0.51) could be confirmed. Statistically, this difference can also be verified, *t* (581) = 8.78, *p* < 0.001, *d* = 0.36. The same applies to the associated facets of compassion, respectfulness, and trust (all *p*-values < 0.001 and all *d*’s > 0.24).

Finally, the assumption that emotional stability is also more pronounced in amateur referees (M = 2.22, SD = 0.54, CI [2.17, 2.26]) compared to the general population (M = 2.66, SD = 0.68) could be descriptively confirmed. Statistically, the difference is also clearly visible, *t* (581) = −19.76, *p* = < 0.001, *d* = −0.82 and is also reflected in the facets of anxiety, depression, and emotional volatility (all *p*-values < 0.001 and all *d*’s > −0.51).

## Discussion

The present study aims at expanding the existing knowledge about the personality profile of handball referees and to learn more about the personality of amateur referees. For this purpose, data from amateur handball referees were collected for the first time and compared with data from expert referees and the German general population. The personality profile of handball referees that emerged in this study is discussed in the following with reference to comparable studies. In addition, the observations are interpreted and placed in the context of the job profile of the handball referee.

With regard to the domain of open-mindedness and the associated facets, there were no conspicuous findings in the comparison between amateur and expert referees. This is consistent with the findings of Gomà-i-Freixanet et al. (2020), who also found no difference in basketball referees from different performance levels. Also with regard to norm values of the German general population, amateur referees seem to be “quite normal people.” Thus, open-mindedness does not seem to be a predictor of performance, unlike what has been suggested for athletes (e.g., Allen et al., 2011; Piedmont et al., 1999). Rather, the results indicate that a particular expression of open-mindedness, including aesthetic sensitivity (interestingly, this score is lower among referees compared to the general population), intellectual curiosity, and creative imagination, is not necessary for the exercise of refereeing. These characteristics could explain why handball referees feel comfortable with familiar territories, tend to be more practical, and do not perceive repetitive activities as boring but, on the contrary, enjoy the routine of a matchday. Moreover, finding one’s own refereeing style takes a lot of practice, but ultimately gives the referee a sense of security. Considering new ways of doing something, e.g., the communication with the referee partner or changing constant routines or rituals, could cause this feeling of security to alter.

In the domain of conscientiousness including the associated facets, it can be observed that amateur handball referees display medium to high values that are almost or even identically equal to the values of expert referees. This contradicts the findings of Gomà-i-Freixanet et al. (2020), who found lower conscientiousness values in lower-ranked basketball referees. In relation to the general population, the referees’ scores can be interpreted as average. This finding is also contrary to study results of basketball referees (Pla-Cortés et al., 2015), who showed significantly lower values than the general population. At the level of facets, the facet responsibility was very striking. It was strongly pronounced in both referee groups and clearly stood out from the value of the general population. Responsibility, therefore, seems to be a personality trait that is essential for handball referees at all levels of performance. Amateur referees must also fulfil their duties, which means that they must first and foremost whistle the matches assigned to them and attend regular further training. Furthermore, they have a role model function which they have to live up to, for example by always being punctual and well prepared. A high degree of conscientiousness could also explain why there are colleagues among amateur referees who manage to consolidate their energy, time, and resources over a long period of time and referee matches well into old age.

In addition, amateur handball referees scored a noteworthy value in the domain of extraversion, which is, however, statistically lower than the value of expert referees. Such a difference could not be observed by Gomà-i-Freixanet et al. (2020) for basketball referees from lower and higher performance levels. Nevertheless, although amateur handball referees display a lower score than expert referees, the value can still be considered high compared to the general population. This underlines the importance of a strongly extroverted personality, reflected in above-average scores for sociability, assertiveness, and energy level among both amateurs and experts. Surprisingly, Pla-Cortés et al. (2015) could not prove this relevance for basketball referees. Looking more closely at the facet level, special attention should be paid to the facet of assertiveness. It is particularly noteworthy that there are only marginal differences between amateur and expert referees, whereas the general population is significantly less assertive. This result stands in conflict with the findings of Gomà-i-Freixanet et al. (2020) and McCarrick et al. (2019), who suggested that higher-ranked referees are more assertive than lower-ranked referees. For amateur referees and handball referees in general, assertiveness, therefore, seems to be an essential characteristic. Even if games in the amateur leagues are not as fast and dynamic as games in the top leagues, the course of the game is still sometimes similarly contested. This implies that even amateur referees must demonstrate assertiveness to effectively employ and maintain match control and credibility. In addition, amateur referees are also engaged in children’s and youth matches, which requires a more pedagogical way of officiating. They have to go up to these young players, be able to talk to them and explain the situation or their decision.

In the domain of agreeableness, amateur referees also scored a considerably high value. This also applied to the facets of compassion and respectfulness. Overall, the values of both the domain and the facets did not differ significantly from the values of the expert referees, which is similar to the findings for basketball referees (Gomà-i-Freixanet et al., 2020). The classification in relation to the norm sample shows that the general population has lower values both in the domain and in all facets. Compared to basketball referees (Pla-Cortés et al., 2015), handball referees are generally characterized by a more compassionate, respectful, and trustworthy nature. Referees, players, and coaches alike share a passion for the sport of handball. The commitment and dedication associated with the numerous hours spent on the activity of officiating described by Fowler, Smith, Nordstrom, and Ferguson (2019) illustrate the intrinsic motivation that keeps referees in the officiating profession. The feeling of sporting togetherness among the referees on the one hand, and the predominantly respectful and trusting environment in handball on the other, create an environment for all involved in which everyone can experience sporting enjoyment and fulfilment.

In the last Big Five domain, negative emotionality, amateur handball referees scored low values both in the domain and in the associated facets of anxiety, depression, and emotional volatility. The expressions did not differ from referees at the expert level, which is consistent with findings for basketball referees (Gomà-i-Freixanet et al., 2020). Furthermore, it is particularly noticeable that handball referees score significantly lower than the general population. This suggests that emotional stability (being less anxious and less depressed) seems to be of fundamental relevance, regardless of performance level. On this point, handball referees differ from athletes, because studies have shown that elite athletes are more emotionally stable than recreational-level athletes (e.g., Allen et al., 2011; Egloff & Gruhn, 1996; Steca et al., 2018). From another perspective, findings from mental health research suggest that depression and anxiety can often be like the flip sides of the same coin, as being depressed often causes anxiety, and anxiety, in turn, can lead to depression. Research also shows that working out and other forms of physical activity can ease symptoms of depression or anxiety and make you feel better (Kalin, 2020). The low values for this domain could, therefore, be due to the fact that referees are not as susceptible to anxiety and depression because of their sporting activities. Apart from this positive side effect of sporting engagement, however, emotional stability is just as important for amateur referees as it is for experts, as they too have to react calmly and unaffectedly to stress in difficult situations.

In summary, it can be stated that amateur referees do not differ significantly from expert referees. Exceptions are the domain of extraversion with the facets of sociability and energy level as well as the facet of aesthetic sensitivity (belongs to the domain of open-mindedness). In relation to the general population, extraversion, particularly with the facet of assertiveness, and negative emotionality (emotional stability) with the facets of anxiety, depression, and emotional volatility, as well as the facet of responsibility (belongs to the dimension of conscientiousness) appear to be important characteristics for handball referees, regardless of the level of performance.

In contemporary research, personality is most commonly measured within the Big Five framework. However, two fundamental dimensions have also loomed large in psychology (Abele & Wojciszke, 2007). Digman (1997) and DeYoung, Peterson, and Higgins (2002) have found in various studies that there are correlations between the five factors of the Big Five and two metatraits. In the context of an integrative interpretation of the results of this study, these two dimensions agency (A) and communion (C; Bakan, 1966) provide indications of why typical patterns often emerge in the Big Five. Within the A–C framework, there are clear links between the Big Two and more specific factors (domains and facets) from the five-factor model. Accordingly, emotional stability, extraversion, and conscientiousness are suggested to be closely related to agency (Abele et al., 2016). In addition, assertiveness is most strongly associated with emotional stability and represents the core component of agency. Applying this to the results of this study, both amateur referees and referees at the expert level can be assigned to the agentic type because of their specific expression in these characteristics. This type is characterized by qualities relevant to goal attainment, such as being ambitious or capable, which is highly applicable to the job profile of referees.

In the context of the group comparisons conducted in this study, the problem of effect sizes should be addressed. This study used *d*_*Cohen*_ with pooled standard deviation to estimate the magnitude of effect between the two referee groups. However, effect sizes in the social sciences, as in the present study, can often be very small (Rosnow & Rosenthal, 2003) which leads to difficulties in interpretation. This is aggravated by the fact that there is no agreement on what magnitude of effect is necessary to establish practical significance (Ferguson, 2009). The guideline suggested by Cohen (1988) is, therefore, understood as a minimum cut-off which means that effect sizes exceeding this cut-off (small effect size; *d* = 0.2) should be interpreted with caution. For this reason, it is not entirely certain for this study whether the differences found (e.g., extraversion: *d* = 0.27) are practically significant.

With reference to the small effect sizes, a cautious interpretation of the results therefore leads to the conclusion that amateur referees and expert referees are almost similar across the BFI‑2 domains and facets. This finding may not seem spectacular at first glance, but it could have directional implications for supporting elements in the refereeing system. As training elements in basic training are becoming more and more standardized, the results of the present study could provide starting points for an integrated psychological coaching concept. Governing bodies in handball should be open to the emerging potentials and feel encouraged to specifically include derivations from and effects of personality in their coaching measures., e.g., for conflict management, emotional control, or coping strategies, or more effective communication and interaction with players and coaches.

The strengths of the present study are a clear focus on amateur referees and a large sample size. In addition, the study creates awareness of the personality traits of handball referees in general and illustrates the applied relevance of personality research in handball refereeing. However, despite the gain in knowledge about the hitherto unexplored personality of amateur referees, this study has some limitations. As described in the “Methods” section, this study only examined male referees. This focus was set because in the reference group (referees at expert level), only male referees were interviewed, making the results more comparable. Nevertheless, female referees represent an equally important target group, not least due to the efforts of the governing bodies to promote female referees. Future studies should, therefore, be encouraged to take a closer look at this group. Finally, we acknowledge that this study was designed as a cross-sectional study and, therefore, does not allow any statements to be made as to whether the personality traits identified were already developed before entering the refereeing profession (gravitational hypothesis) or whether they only developed over the years in the profession (change hypothesis). For this reason, we recommend conducting long-term studies to identify whether personality directly contributes to the decision to become a referee (selection effect) or whether refereeing activity contributes to personality development (socialization effect).

The results of this study provide extended insights into the personality profile of handball referees, which could lay the foundation for further research. This should address the recruitment, retention, or education of referees. With the number of referees decreasing and the COVID-19 pandemic exacerbating this negative trend, the biggest challenge is recruitment of referees. The difficulty usually lies in attracting potential candidates in the first place. The job of the referee is often perceived as a heavy burden and there is uncertainty on the part of the candidates as to whether they are “suitable” for this job at all. In order to at least reduce the concern about the suitability of one’s own personality, personality assessments could provide orientation and thus tip the scales in favor of entering the refereeing career. Once the referees are in the system, increased awareness of their personality traits can help them better manage these attributes during performance (Cunningham, Simmons, Mascarenhas, & Redhead, 2014). This reinforces the need for objective assessment methods for assessing the personality types of referees when recruiting, developing, and evaluating referees.

We hope that we have succeeded in arousing the interest of sports psychology researchers and handball authorities and in demonstrating the various possible applications of personality research in handball refereeing. We are convinced that the results of the present study will support the progression of this field.

## Appendix

﻿

**Table 1:** Descriptive and Inferential Statistics of the Big Five Inventory 2

	Amateur referees (*n* = 582)			Expert referees (*n* = 163)			*Df*	*t*	*p-*value	*d*
	M	SD	95% CI	M	SD	95% CI				
*Open-Mindedness*	3.26	0.60	[3.21, 3.31]	3.16	0.62	[3.07, 3.26]	743	1.77	0.077	0.15
Aesthetic Sensitivity	2.59	0.95	[2.51, 2.67]	2.39	0.97	[2.24, 2.55]	743	2.31	0.021	**0.21**
Intellectual Curiosity	3.66	0.68	[3.60, 3.71]	3.60	0.70	[3.49, 3.71]	743	0.99	0.321	0.08
Creative Imagination	3.52	0.75	[3.46, 3.59]	3.49	0.74	[3.38, 3.61]	743	0.43	0.668	0.04
*Conscientiousness*	3.72	0.64	[3.67, 3.78]	3.72	0.57	[3.63, 3.81]	743	0.02	0.984	0.00
Organization	3.67	0.93	[3.59, 3.74]	3.68	0.83	[3.56, 3.81]	743	−0.23	0.818	−0.02
Productiveness	3.55	0.74	[3.49, 3.61]	3.49	0.68	[3.39, 3.60]	743	0.76	0.447	0.07
Responsibility	3.96	0.56	[3.91, 4.00]	3.98	0.52	[3.90, 4.06]	743	−0.55	0.582	−0.05
*Extraversion*	3.53	0.55	[3.48, 3.57]	3.67	0.56	[3.59, 3.76]	743	−2.97	0.003	**−0.26**
Sociability	3.40	0.78	[3.34, 3.46]	3.62	0.81	[3.50, 3.76]	743	−3.29	0.001	**−0.29**
Assertiveness	3.73	0.64	[3.68, 3.78]	3.78	0.59	[3.69, 3.88]	743	−1.06	0.289	−0.09
Energy Level	3.45	0.62	[3.40, 3.50]	3.60	0.60	[3.51, 3.70]	743	−2.76	0.006	**−0.24**
*Agreeableness*	3.81	0.45	[3.77, 3.84]	3.86	0.41	[3.80, 3.93]	743	−1.45	0.147	−0.13
Compassion	3.92	0.59	[3.87, 3.97]	4.00	0.57	[3.91, 4.09]	743	−1.56	0.119	−0.15
Respectfulness	4.19	0.53	[4.15, 4.23]	4.22	0.49	[4.15, 4.30]	743	−0.79	0.428	−0.07
Trust	3.30	0.61	[3.26, 3.35]	3.35	0.55	[3.27, 3.45]	743	−1.02	0.308	−0.09
*Negative Emotionality*	2.22	0.54	[2.17, 2.26]	2.16	0.51	[2.08, 2.24]	743	1.14	0.254	0.10
Anxiety	2.45	0.63	[2.40, 2.50]	2.34	0.60	[2.25, 2.44]	743	1.86	0.063	0.16
Depression	2.00	0.63	[1.95, 2.05]	1.92	0.56	[1.83, 2.00]	743	1.53	0.128	0.14
Emotional Volatility	2.20	0.71	[2.14, 2.26]	2.22	0.68	[2.12, 2.33]	743	−0.35	0.726	−0.03

Effect sizes > 0.20 are in boldface

*CI* confidence interval, *SD *standard deviation, *M *mean

**Table 2:** Descriptive and Inferential Statistics of the Big Five Inventory 2

	Amateur referees (*n* = 582)			General population (*n* = 613)		*Df*	*t*	*p*-value	*d*
	M	SD	95% CI	M	SD				
*Open-Mindedness*	3.26	0.60	[3.21, 3.31]	3.28	0.63	581	−0.87	0.385	−0.04
Aesthetic Sensitivity	2.59	0.95	[2.51, 2.67]	2.89	0.96	581	−7.53	< 0.001	−0.31
Intellectual Curiosity	3.66	0.68	[3.60, 3.71]	3.46	0.71	581	7.04	< 0.001	0.29
Creative Imagination	3.52	0.75	[3.46, 3.59]	3.50	0.76	581	0.77	0.444	0.03
*Conscientiousness*	3.72	0.64	[3.67, 3.78]	3.57	0.59	581	5.71	< 0.001	0.24
Organization	3.67	0.93	[3.59, 3.74]	3.60	0.85	581	1.75	0.081	0.07
Productiveness	3.55	0.74	[3.49, 3.61]	3.44	0.70	581	3.45	< 0.001	0.14
Responsibility	3.96	0.56	[3.91, 4.00]	3.66	0.57	581	12.61	< 0.001	**0.52**
*Extraversion*	3.53	0.55	[3.48, 3.57]	3.13	0.61	581	17.35	< 0.001	**0.72**
Sociability	3.40	0.78	[3.34, 3.46]	3.05	0.78	581	10.90	< 0.001	0.45
Assertiveness	3.73	0.64	[3.68, 3.78]	3.21	0.73	581	19.60	< 0.001	**0.81**
Energy Level	3.45	0.62	[3.40, 3.50]	3.11	0.68	581	13.33	< 0.001	**0.55**
*Agreeableness*	3.81	0.45	[3.77, 3.84]	3.64	0.51	581	8.78	< 0.001	0.36
Compassion	3.92	0.59	[3.87, 3.97]	3.78	0.66	581	5.77	< 0.001	0.24
Respectfulness	4.19	0.53	[4.15, 4.23]	4.00	0.60	581	8.66	< 0.001	0.36
Trust	3.30	0.61	[3.26, 3.35]	3.15	0.64	581	6.17	< 0.001	0.26
*Negative Emotionality*	2.22	0.54	[2.17, 2.26]	2.66	0.68	581	−19.76	< 0.001	**−0.82**
Anxiety	2.45	0.63	[2.40, 2.50]	2.84	0.71	581	−15.19	< 0.001	**−0.63**
Depression	2.00	0.63	[1.95, 2.05]	2.56	0.83	581	−21.47	< 0.001	**−0.89**
Emotional Volatility	2.20	0.71	[2.14, 2.26]	2.56	0.79	581	−12.19	< 0.001	**−0.51**

Effect sizes > 0.50 are in boldface

*CI* confidence interval, *SD *standard deviation, *M *mean

**Table 3:** Confidence intervals of the mean difference

	95% CI	
	*LL*	*UL*
*Open-Mindedness*	−0.20	0.01
Aesthetic Sensitivity	**−0.36**	**−0.03**
Intellectual Curiosity	−0.18	0.06
Creative Imagination	−0.16	0.10
*Conscientiousness*	−0.11	0.11
Organization	−0.14	0.18
Productiveness	−0.18	0.08
Responsibility	−0.07	0.12
*Extraversion*	**0.05**	**0.24**
Sociability	**0.09**	**0.37**
Assertiveness	−0.05	0.17
Energy Level	**0.04**	**0.26**
*Agreeableness*	−0.02	0.14
Compassion	−0.02	0.18
Respectfulness	−0.05	0.13
Trust	−0.05	0.16
*Negative Emotionality*	−0.15	0.04
Anxiety	−0.21	0.01
Depression	−0.19	0.02
Emotional Volatility	−0.10	0.15

*CI* confidence interval, *LL* lower limit, *UL* upper limit

**Table 4:** Reliability coefficients and intercorrelations of the Big Five Inventory 2 facets

Model		Open-Mindedness			Conscientiousness			Extraversion			Agreeableness			Negative Emotionality	
	α	Aes	Int	Cre	Org	Pro	Respo	Soc	Ass	Ene	Com	Respe	Tru	Anx	Dep
Aesthetic Sensitivity	0.82	–	–	–	–	–	–	–	–	–	–	–	–	–	–
Intellectual Curiosity	0.60	0.42**	–	–	–	–	–	–	–	–	–	–	–	–	–
Creative Imagination	0.81	0.24**	0.41**	–	–	–	–	–	–	–	–	–	–	–	–
Organization	0.87	0.05	−0.02	0.00	–	–	–	–	–	–	–	–	–	–	–
Productiveness	0.73	0.05	0.08	0.15**	0.65**	–	–	–	–	–	–	–	–	–	–
Responsibility	0.63	0.04	0.09*	0.05	0.57**	0.64**	–	–	–	–	–	–	–	–	–
Sociability	0.77	0.02	0.07	0.23**	0.04	0.11**	0.01	–	–	–	–	–	–	–	–
Assertiveness	0.76	0.03	0.25**	0.30**	0.08	0.19**	0.13**	0.50**	–	–	–	–	–	–	–
Energy Level	0.65	0.22**	0.22**	0.38**	0.23**	0.34**	0.18**	0.55**	0.42**	–	–	–	–	–	–
Compassion	0.64	0.21**	0.15**	0.19**	0.09*	0.10*	0.18**	0.14**	0.05	0.24**	–	–	–	–	–
Respectfulness	0.65	0.06	0.04	0.04	0.22**	0.25**	0.32**	0.07	−0.12**	0.11*	0.42**	–	–	–	–
Trust	0.57	0.10*	0.10*	0.08	0.02	0.06	0.09*	0.03	−0.13**	0.15**	0.43**	0.46**	–	–	–
Anxiety	0.60	−0.09*	−0.16**	−0.12**	−0.07	−0.22**	−0.26**	−0.09*	−0.27**	−0.17**	0.00	−0.25**	−0.18**	–	–
Depression	0.79	−0.09*	−0.21**	−0.17**	−0.21**	−0.35**	−0.32**	−0.28**	−0.29**	−0.41**	−0.15**	−0.28**	−0.23**	0.56**	–
Emotional Volatility	0.78	−0.12**	−0.12**	−0.06	−0.18**	−0.28**	−0.32**	0.11**	0.03	−0.10*	−0.12**	−0.48**	−0.32**	0.57**	0.45**
*Mean*	0.71	–	–	–	–	–	–	–	–	–	–	–	–	–	–

*N* = 582

**p* < 0.05

***p* < 0.01

*Aes* Aesthetic Sensitivity, *Int* Intellectual Curiosity, *Cre* Creative Imagination, *Org* Organization, *Pro* Productiveness, *Respo* Responsibility, *Soc* Sociability, *Ass* Assertiveness, *Ene* Energy Level, *Com* Compassion, *Respe* Respectfulness, *Tru* Trust, *Anx* Anxiety, *Dep* Depression

## Footnotes

1 Compared to Dodt et al. (2022), the referees in this study were older and had a longer career. In contrast, they had a lower level of educational attainment and a lower fitness level.

2 If a 95% confidence interval includes the null value, then there is no statistically meaningful or statistically significant difference between the groups. If the confidence interval does not include the null value, then we conclude that there is a statistically significant difference between the groups.

## Declarations

All procedures performed in studies involving human participants or on human tissue were in accordance with the ethical standards of the institutional and/or national research committee and with the 1975 Helsinki declaration and its later amendments or comparable ethical standards. We obtained the approval of the local ethics committee (ethics application no. 082/2020). Informed consent was obtained from all individual participants included in the study.
